# Promising short-term outcomes of free-hand burring technique to implant second-generation metaphyseal cone in Asian knees – a case series

**DOI:** 10.1186/s42836-024-00254-2

**Published:** 2024-07-02

**Authors:** Thomas Ka Chun Leung, Ping Keung Chan, Henry Fu, Amy Cheung, Michelle Hilda Luk, Lawrence Chun Man Lau, Kwong Yuen Chiu

**Affiliations:** 1 Department of Orthopaedics and Traumatology, Queen Mary Hospital, Hong Kong SAR, China; 2 Department of Orthopaedics and Traumatology, School of Clinical Medicine, The University of Hong Kong, Hong Kong SAR, China

**Keywords:** Metaphyseal cone, Second-generation cone, Bone defect, Revision knee arthroplasty, Asian knee

## Abstract

**Background:**

The second-generation metaphyseal cone was useful in managing bone defects in revision knee arthroplasty. However, due to the anatomical constraints in Asian osteometry, the authors utilized a novel free-hand burring technique instead of cannulated reaming for bone preparation. We reported the short-term outcomes of our surgical techniques specific to Asian osteometry.

**Methods:**

We conducted a case series by consecutively recruiting 13 female and 12 male patients (involving 25 knees), with a mean age of 71 years (range, 54–88 years). The patients underwent revision total knee arthroplasty during the period from April 2017 to June 2022. Twenty-three tibial cones and 4 femoral cones using free-hand burring technique were implanted. The mean follow-up duration was 51 months (range 18–80 months). Due to the relatively small bone size and meta-diaphyseal center mismatch in the Asian knees, the free-hand burring technique instead of the cannulated reaming technique was adopted in preparing for cone implantation. The clinical outcomes were knee ranges of motion, the Knee Society Knee scores (KSS), end-of-stem pain, infection, and the need for revision surgery. The radiological outcomes included osteointegration, fracture, and loosening.

**Results:**

Mean knee range of motion improved from 83 degrees (range 0°–120°) preoperatively to 106 degrees (range 60°–125°) postoperatively (*P* < 0.001). Mean KSS improved significantly from 29 (range 0–70) to 69 (range 5–100) (*P* < 0.001). All cones were osteointegrated. One case had transient end-of-stem pain, two developed intraoperative minor femoral fractures and one suffered from recurrent infection that did not require cone revision. Cone revision-free survivorship was 100%. There was no aseptic loosening.

**Conclusions:**

The second-generation cone implanted with free-hand burring bone preparation yielded promising short-term outcomes in Asian knees.

## Background

The demand for revision total knee arthroplasty (TKA) is on the rise with the aging population [1]. Management of bone loss in revision cases can be challenging. The Anderson Orthopaedic Research Institute (AORI) classification system is commonly adopted to classify bone defects [2]. Metaphyseal cone is useful in filling in the bone defect and improving metaphyseal fixation of the prosthesis, in compliance with the concept of zonal fixation [3].

The first-generation cone, despite the reported satisfactory outcomes, was associated with surgical difficulties in bony preparation by free-hand burring, which posed technical challenges and potential inaccuracy in conforming to the bone defect and osteometry. The recent advent of the second-generation cone is manufactured by three-dimensional printing according to a computed tomography-based anatomical database of a diverse population. It is made up of porous titanium to fit the geometry of the native tibia and femur. Bone preparation is streamlined into a cannulated-reaming system with precise sizing and morphology. It is relatively user-friendly and provides standardized bone preparation for implantation. From a biomechanical perspective, the new titanium cone has been verified to be non-inferior or even slightly superior to tantalum in withstanding physiological loading, with equal or fewer micromotions upon cyclical loading [4].

Only a limited number of European studies have been published to show encouraging short-term outcomes of second-generation titanium cone in revision TKA [5–10]. To the authors’ best knowledge, such data on the Asian population are still lacking. Therefore, we aimed to study the clinical and radiological outcomes of second-generation titanium cones in our Asian locality.

## Methods

**Fig. 1:**
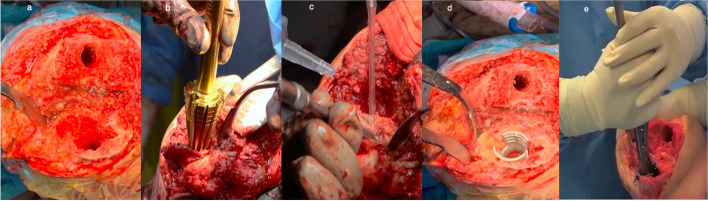
(**a**) AORI class 2B bone defect after implant extraction; (**b**) Cannulated reamer misguided onto anterior cortex using intramedullary guide; (**c**) Free-hand burring technique in bone preparation; (**d**) Cone impacted; (**e**) Cone stability tested by lifting the leg using the cone holder

We reviewed 25 patients (13 females and 12 males, with 25 knees involved), who underwent revision total knee arthroplasty in our center during the period from April 2017 to June 2022. Our study was approved by the Institutional Review Board of The University of Hong Kong/Hospital Authority Hong Kong West Cluster, with the reference number UW 20-253. Informed consents were obtained from the patients, including consent for publication of the images in Figs. 1 and 4. Inclusion criteria were revision TKA using tibial and/or femoral second-generation titanium metaphyseal cones (Triathlon Tritanium Cone, TS Revision Knee System, Stryker, Mahwah, NJ, USA), implantation of which was prepared by free-hand burring techniques due to the anatomical constraints of the knees that precluded the use of cannulated reamer. Follow-up intervals were postoperative 2 weeks, 6 weeks, 3 months, 6 months then a year. The minimum follow-up duration lasted for 18 months, and the mean follow-up duration was 51 months (range 18–80 months).

Demographic data including age, gender, indication, and AORI classification were noted. Clinical outcomes recorded at the latest follow-up included knee range of motion, end-of-stem pain, Knee Society Knee Scores (KSS), infection, and any need for revision surgery. Osteointegration was assessed on serial postoperative radiographs, which were also scrutinized for any complications, including fracture, radiolucency, and aseptic loosening.

All revision TKA were performed by a single senior chief surgeon. The standard medial parapatellar approach was adopted in all cases. Implants were removed with maximal bone stock preservation. The bone defect was classified intraoperatively following implant removal, using the AORI classification (Fig. 1a). Class IIA and IIB defects were considered indicated for cone augmentation. No bone graft was used as cone was considered adequate in achieving metaphyseal structural support to allow initial mechanical stability for early weight-bearing. The tibia cut surface was assessed first to decide whether recut was needed, followed by the femoral side. Flexion and extension gap balancing were measured with the adjustable spacer block to help determine the appropriate insert thickness and joint line. Femoral rotation was determined with reference to gap balancing method. The joint line was restored, with femoral component distalization and augment in some cases.

In preparing for cone implantation, cannulated reaming according to the second-generation cone manual was considered contraindicated in the included cases due to the anatomical mismatch between the metaphyseal center and diaphyseal center in those Asian knees. The reamer was guided directly onto the anterior tibial cortex in some cases (Fig. 1b). Instead, the free-hand burring technique was adopted for bone preparation in all cases, similar to the preparation for the first-generation cone (Fig. 1c). Steps of our surgical techniques were detailed as follows. An appropriately sized cone was chosen based on the bone defect and bone size. The proximal contour of the cone was marked on the proximal metaphyseal bone surface, followed by the depth and distal contour, according to the size of the cone. Using a 5-mm tip high-speed burr, the proximal bone surface was prepared in a centrifugal manner to match the marking. The bone was burred gradually from proximal to distal in order to match the contour of the cone (Fig. 1c). It was important to achieve press-fit by gradually increasing the burring surface radially outwards until the stability was tested satisfactory as indicated by the leg being lifted using a cone holder holding onto the implanted cone (Fig. 1d–e). The cone was considered stable if the leg could be lifted off the table with the cone holder without loosening the cone. If stability was inadequate, repeated burring and upsizing of the cone would be performed until adequate press-fit is achieved.

**Fig. 2:**
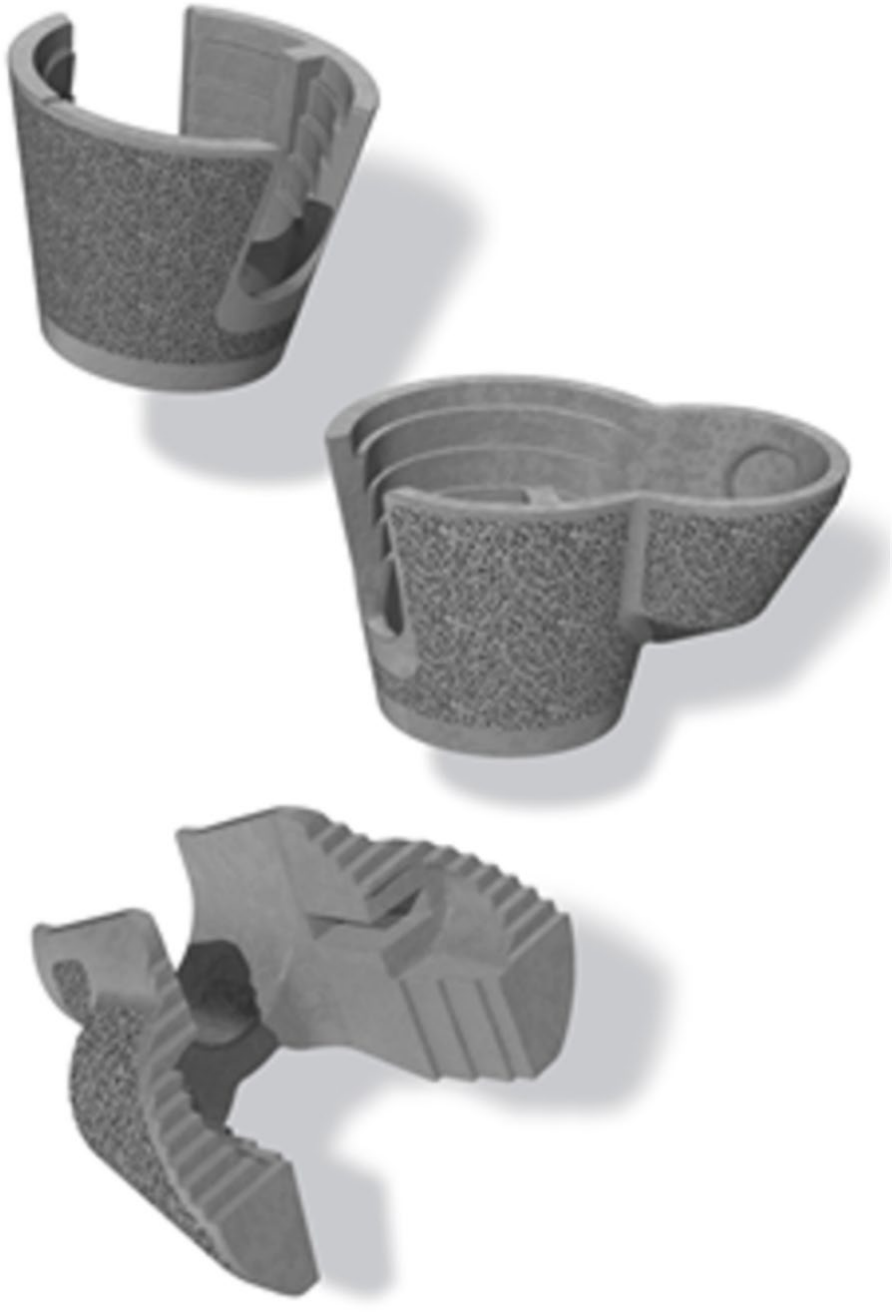
Triathlon TS cones, Stryker Triathlon Revision Knee System Protocol

Triathlon revision knee system was implanted in conjunction with the cones in all cases (Fig. 2). Subsequently, mediolateral laxities were tested following trial implant insertion. Varus-valgus constrained implants with cemented stems were utilized in cases with significant residual mediolateral laxity, with their reported excellent mid-term survivorship [10]. Cone was then impacted to achieve press-fit, with satisfactory intraoperative axial and rotational stability. Stemmed knee prostheses were then cemented to the inner surface of the implanted cone, using antibiotics-loaded cement, ensuring joint line restoration. There was no need for offset stem in our case series. We used relatively small short stems to be cemented onto the implanted cones, allowing for adjustment to accommodate any meta-diaphyseal center mismatch.

Postoperatively, all patients were allowed to engage in immediate weight-bearing (full weight-bearing except for two cases with femoral fractures which were allowed protected weight-bearing for six weeks then full weight-bearing). All patients followed a standardized multidisciplinary rehabilitation protocol.

## Results

A total of 27 cones were used in 25 knees of 25 patients (13 females and 12 males) receiving revision knee arthroplasty. The indications for revision knee arthroplasty were periprosthetic joint infection (13 out of 25), aseptic loosening (9 out of 25). The indications of the other 3 cases included postoperative knee stiffness, insert dislocation and PE wear. The mean age was 71 (range 54–88). Among the 27 cones, 23 were tibial and 4 were femoral. Two patients received simultaneous femoral and tibial cones. Of the 23 tibial cones, 18 were symmetrical and 5 were asymmetrical. Regarding the cone sizes, among tibial cones, 9 were size A (the smallest), 7 were size B, 4 were size C, 2 were size D and 1 was size E. All the femoral cones (Table 1) were size 1 & 2 (the smallest).

**Table 1:** Results of the case series

**Age/Sex**	**Side (L/R)**	**Indication**	**FU time (months)**	**Cone type, size**	**AORI class**	**Functional KSS gain**	**Range gain (degrees)**	**Complication**
57/M	L	Loosening	18	Tibia, E	2A	55	5	Nil
54/M	R	Infection	23	Femur, 1–2	2B	35	10	Nil
65/M	L	Infection	24	Tibia, B; Femur, 1–2	2B	45	30	Nil
85/F	R	Loosening	30	Tibia, A	2B	65	30	Nil
59/M	R	Infection	35	Tibia, C	2B	10	0	Nil
65/F	R	Loosening	36	Tibia, A	2B	10	10	Nil
86/F	L	Instability	42	Tibia, A	2B	35	10	Nil
77/M	R	Dislocated insert	43	Tibia, C	2B	80	45	Nil
65/M	L	Loosening	44	Tibia, C	2A	30	0	Nil
62/M	R	Infection	44	Tibia, C	2B	70	60	Nil
73/F	R	Infection	48	Tibia, A	2B	5	20	Nil
61/M	R	Infection	51	Tibia, B	2B	65	60	Nil
65/M	R	Infection	52	Tibia, A	2B	20	25	Nil
71/M	L	Stiffness	54	Tibia, A	2B	35	25	Nil
74/F	L	Infection	55	Tibia, A	2B	5	15	Nil
72/M	L	Infection	60	Tibia, B	2B	0	25	Nil
65/F	L	Infection	62	Tibia, B	2A	10	10	Nil
79/F	R	loosening	64	Tibia, A	2B	25	10	Nil
77/M	L	Loosening	68	Tibia, A	2A	35	50	Nil
55/F	L	Loosening	69	Tibia, B	2B	35	10	Nil
78/F	L	Loosening	70	Tibia, B	2B	25	0	Nil
80/F	L	Infection	72	Tibia, B; Femur, 1–2	2B	85	25	Femoral fracture
79/F	R	Loosening	76	Femur, 1–2	2A	50	0	Femoral fracture
71/F	L	Infection	77	Tibia, D	2A	80	50	Nil
88/F	L	Infection	80	Tibia, D	2B	50	0	Nil

In all cases, the stem was cemented to the inner surface of the cone, with antibiotics-loaded cement. Cemented metaphyseal-engaging stems were utilized in all except one case requiring tibial tubercle osteotomy, in which a hybrid fixation with cemented metaphyseal and cementless diaphyseal-engaging stem was used. The tibial stem length used was 100 mm in 21 out of 25 cases, and 150 mm in the 4 remaining cases. Stem diameter was 12 mm in 15 cases, 10 mm in 6 cases, and 9 mm in 4 cases.

**Fig. 3:**
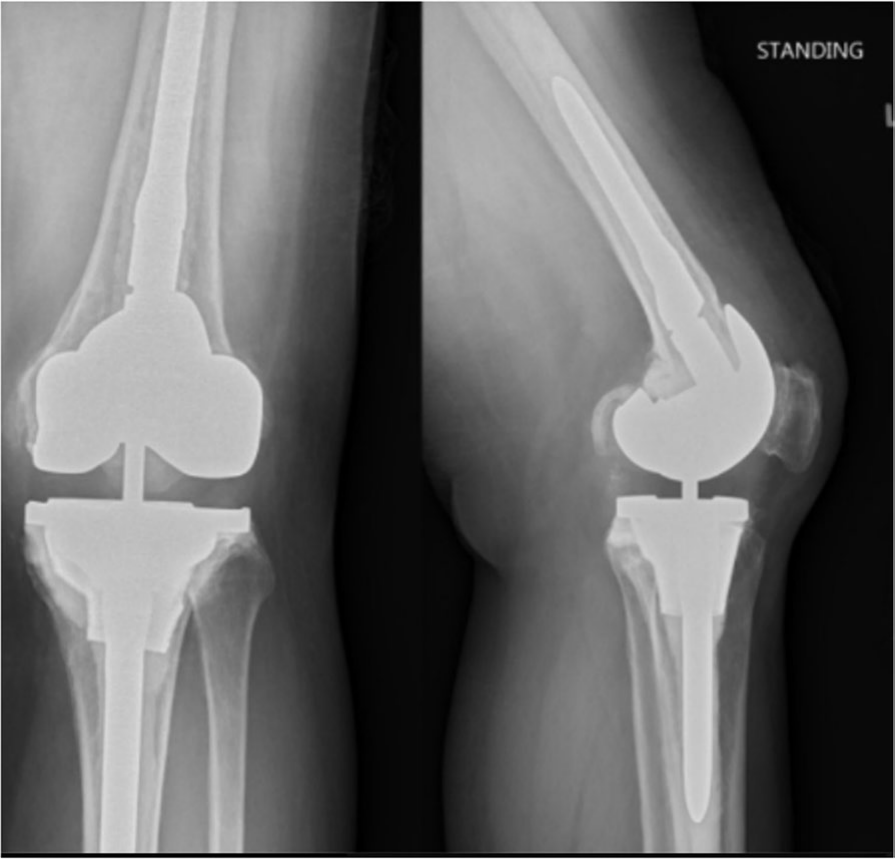
Postoperative radiograph of a case receiving metaphyseal cone using free-hand burring technique

Mean knee range of motion improved from 83 degrees (range 0°–120°) preoperatively to 106 degrees (range 60°–125°) postoperatively (*P* < 0.001). Mean KSS improved significantly from 29 (range 0–70) to 69 (range 5–100) (*P* < 0.001). There was only one transient mild end-of-stem pain in a case with a 150 mm long cemented tibial stem, which resolved upon follow-up. Osteointegration was observed in all knees (Fig. 3).

Two intraoperative fractures occurred at anterior femoral cortices during femoral cone impaction and were treated with cerclage wires and protected weight-bearing. Subsequently, the fractures healed with cone osteointegration. One case required revision with debridement, antibiotics, and insert exchange with the cone retained, to treat the recurrent periprosthetic infection at 1.5 years after the two-stage revision with a tibial cone. No aseptic loosening was noted. Cone revision-free survivorship was 100%. There was one mortality unrelated to orthopaedic conditions at postoperative one year.

## Discussion

The most important outcome of this study was that promising mid-term outcomes could be achieved by using free-hand burring technique to implant second-generation metaphyseal cone, despite the anatomical differences in Asian osteometry which limited the designated cannulated reaming bone preparation.

Several options are available for managing bone defects in revision total knee arthroplasty. For AORI Class I, i.e., contained defects, cement and screws are commonly adopted. With AORI Class IIA/B and III, there is a metaphyseal deficiency that hinders cement interdigitation. The classical technique of bone grafting, be it cancellous or structural allograft, bears its intrinsic risks of non-union, infection, and resorption [12]. Metallic augmentation in the forms of metaphyseal cone and sleeve is gaining popularity in recent years.

These two types of metal implants are based on different mechanisms. Each type of implant has its pros and cons. The sleeve is designed for use in conjunction with a revision prosthesis to improve axial rotational stability in metaphyseal bone defects. It requires sequential broaching to achieve press-fit. It is designed to be implanted with the prosthesis in one piece, and, hence, would not allow for independent adjustment of the sleeve and stem [13, 14]. On the other hand, cone allows independent metaphyseal fixation followed by cementation of the prosthesis inside the implanted cone to allow for more flexible joint line restoration and rotational adjustment [15, 16]. In particular, in our case series, we managed to use short small cemented stems within the cones for the flexibility to adjust for meta-diaphyseal center mismatch, hence not requiring the use of offset stem in all our cases. Both titanium sleeve and tantalum cone have been widely proven effective and durable in the current literature, with low rates of aseptic loosening [17–19].

First-generation tantalum cones have yielded excellent mid-term outcomes, with low rates of loosening and revision in multiple studies [15, 16, 20, 21]. However, the first-generation cones require free-hand burring for preparation, which is considered technically demanding and prone to inaccuracy. Furthermore, its applicability could be limited by the morphology and size of bone, due to a lack of precision in bone preparation and sizing. On the contrary, second-generation titanium metaphyseal cone is designed based on a CT anatomy database, using 3D printing technology [4]. It achieves technical efficiency by its designated cannulated reaming preparation, with improved accuracy in sizing and press-fit. These are technically advantageous to the surgeon as it allows streamlined preparation and accurate press-fit, which are lacking in the first-generation cone. The advancement of the second-generation cone has the theoretical potential to sharpen the biomechanical advantage of the previous generation while reducing the technical difficulty. However, only short-term results have been reported in the scarce literature (Table 2) [5–10]. Cemented stems were used in the vast majority of our cases as they allowed minor adjustments of implant position, potentially reduced end-of-stem pain, and minimized the risk of fracture as compared to cementless long stems, with no differences in loosening rate [11, 22].

**Table 2:** Summary of existing publications on short-term outcomes of second-generation cone

**Author**	**Year**	**Patient No.**	**Cone No.**	**Mean follow-up duration (Months)**	**Aseptic loosening rate**	**Rate of revision for aseptic loosening**	**Survivorship free of cone revision**
Patel et al. [5]	2016	3	3	5	0	0	100%
Denehy et al. [6]	2019	62	77	27	0	0	90.2%
Tetreault et al. [7]	2020	139	202	29	3.3%	0	98%
Remily et al. [8]	2021	54	54	24 (minimum)	1.5%	1.5%	88.2%
Chalmers et al. [9]	2021	163	163	30	0	0	96%
Monárrez et al. [10]	2022	62	62	48	3%	3%	96%
Leung et al.	2024	25	27	51	0	0	100%

Tetreault et al. reported one of the largest case series of 139 patients with 202 second-generation cones, yielding an excellent 2-year survivorship with 100% survivorship free of revision for aseptic loosening and 98% survivorship without cone revision [7]. Similarly, Chalmers et al. also reported, in their large series of 163 patients, an excellent 2-year survivorship without cone revision of 96%, and a survivorship free of revision for aseptic loosening of 100% [9]. Monárrez et al. reported a 96% overall cone revision-free survivorship in their 62 patients over a mean follow-up period of 2 years. [10] These studies verified the applicability of cannulated reaming preparation of cone in Caucasian patients with excellent short-term outcomes.

Our study was the first to report the implications of Asian osteometry on the surgical techniques for preparing for the second-generation titanium metaphyseal cone. First, the relatively small-sized tibial and femoral canals would accommodate only the smallest one to two sizes of implants for the majority of our cases. It could potentially predispose the bone to fracture during cone preparation and impaction as a result of bone-implant size mismatch. Furthermore, it is compounded by femoral and tibial bowing. Tang et al. and Yau et al. have respectively proven tibial and femoral bowing in both sagittal and coronal planes in Asian knees [23, 24]. Tang et al. found significant sagittal plane femoral bowing in the distal third of the femora in their radiographic study of 100 lower limbs of Chinese patients [23]. Yau et al., in their radiographic study of 92 Chinese lower limbs, reported significant coronal plane bowing, defined as more than 2 degrees, in 62% of the femurs, and 32% of the tibias [24]. These were considered risk factors for mal-alignment and fractures, hence necessitating long films for preoperative planning.

**Fig. 4:**
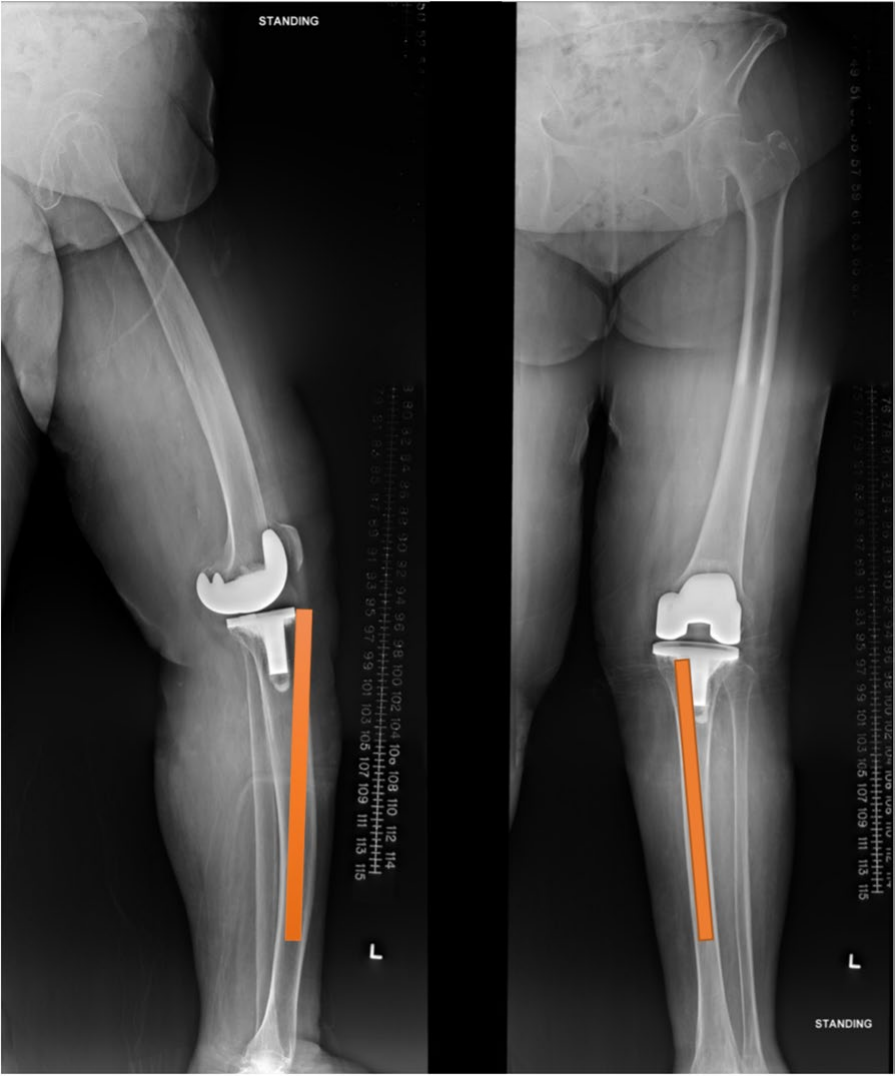
XRs illustrating metadiaphyseal mismatch common in Asian knee

Moreover, meta-diaphyseal center mismatch was reportedly more prevalent in the Asian population, in which the center of tibia diaphysis is often offset from the metaphyseal center. In a more recent CT cross-sectional study by Tang et al., the center of the tibial plateau and the center of the tibia shaft were defined by locating the centers of the best-fitting rectangle and circle respectively in axial CT images, based on standardized anatomical landmarks. They concluded that in Chinese patients, the tibial shaft center was commonly anterolateral to the tibial plateau center, with the offset being more pronounced in male than female patients [25]. Our experience concurred with the phenomenon of meta-diaphyseal center mismatch found in the Asian population. As depicted in Fig. 4, the diaphyseal intramedullary guiding rod would offset the cannulated reamer at the metaphyseal level, due to the anterolateral eccentric position of the diaphyseal center with reference to the metaphyseal center, thereby predisposing bone to cortical abutment or fracture on reaming. It should be noted that the designated cannulated reaming preparation was utilized in virtually all the reported Caucasian series. Our study was the first to report the free-hand burring technique in preparing for the second-generation cone, with promising mid-term outcomes commensurate with the reamer-prepared cone results in Caucasians. Remarkably, there was no aseptic loosening in our series, with a 100% cone revision-free survivorship. Our findings are valuable and clinically-pertinent, especially for Asian patients given their different osteometry.

Further studies incorporating computed tomography are needed to delineate the exact anatomical differences between Asian and Caucasian knees and to elucidate the local applicability of existing cannulated reaming preparation. This will also shed light on modifications in implants to accommodate Asian osteometry. From the authors’ perspective, it will have impactful potential to expand the applicability of future-generation cones to suit different osteometry and ethnicity, hence translating to improved outcomes.

Our study had the following limitations. First, it was a single-surgeon series which might limit the generalizability of the surgical techniques and results. Second, the anatomical constraints were assessed intraoperatively based on the surgeons’ experience. Ideally, CT-based studies would be valuable in delineating the osteometry, hence dictating which bone preparation technique was of choice. Third, the outcome measurements were on short-term basis only. Longer-term follow-ups are necessary to conclude on the longevity of cone implantation using our surgical techniques.

## Conclusions

Second-generation titanium metaphyseal cone implanted with free-hand burring bone preparation yielded promising mid-term clinical and radiological results in our locality. Our study highlights the anatomical constraints in Asian knees which could potentially limit the applicability of cannulated reaming designated for second-generation cone. Caution should be exercised in preparing bone for cone implantation in Asian patients given their relatively small and bowed bone and meta-diaphyseal center mismatch.

## Data Availability

The datasets used and/or analyzed during the current study are available from the corresponding author upon reasonable request.

## Abbreviations

KSS: Knee Society Knee Scores
TKA: Total Knee Arthroplasty
AORI: Anderson Orthopaedic Research Institute
